# A matched case-control study of early cervical spondylotic myelopathy based on diffusion magnetic resonance imaging

**DOI:** 10.1186/s13244-023-01579-3

**Published:** 2024-01-25

**Authors:** Ming Ni, Shujing Li, Xiaoyi Wen, Ben Wang, Chenyu Jiang, Xianchang Zhang, Ning Lang, Liang Jiang, Huishu Yuan

**Affiliations:** 1 Department of Radiology, Peking University Third Hospital, Beijing, China; 2 Institute of Statistics and Big Data, Renmin University of China, Beijing, China; 3 Department of Orthopedics, Peking University Third Hospital, Beijing, China; 4 Engineering Research Center of Bone and Joint Precision Medicine, Beijing, China; 5 Beijing Key Laboratory of Spinal Disease Research, Beijing, China; 6 MR Collaboration, Siemens Healthcare Ltd., Beijing, China

**Keywords:** Cervical spondylotic myelopathy, Diffusion magnetic resonance imaging, Regression analysis, Surgery

## Abstract

**Background:**

Early cervical spondylotic myelopathy (CSM) is challenging to diagnose and easily missed. Diffusion MRI (dMRI) has the potential to identify early CSM.

**Methods:**

Using diffusion tensor imaging (DTI), diffusion kurtosis imaging (DKI), and neurite orientation dispersion and density imaging (NODDI), a 1:1 matched case-control study was conducted to evaluate the potential of dMRI in identifying early CSM and assessing uncompressed segments of CSM patients. CSM patients and volunteers were matched by age and spinal location. The differences in dMRI parameters between groups were assessed by the paired *t*-test, the multicollinearity of the dMRI parameters was evaluated by the variance inflation factor (VIF), and the value of dMRI parameters in distinguishing controls from CSM patients was determined by logistic regression. The univariate *t*-test was used to analyse differences between CSM patients and volunteers in adjacent uncompressed areas.

**Results:**

In total, 56 CSM patients and 56 control volunteers were included. Paired *t*-tests revealed significant differences in nine dMRI parameters between groups. Multicollinearity calculated through VIF and combined with logistic regression showed that the orientation division index (ODI) was significantly positively correlated (*r* = 2.12, *p* = 0.035), and the anisotropic water fraction (AWF) was significantly negatively correlated (*r* = −0.98, *p* = 0.015). The fractional anisotropy (FA), mean diffusivity (MD), radial diffusivity (RD), isotropic volume fraction (ISOVF), ODI, and AWF were significantly different in the upper and lower uncompressed areas at all ages.

**Conclusion:**

dMRI can noninvasively identify early CSM patients and potentially identify the extent of CSM lesions involving the cervical spinal cord.

**Critical relevance statement:**

Diffusion MRI (dMRI) can identify early cervical spondylotic myelopathy (CSM) and has the potential to help determine the extent of CSM involvement. The application of dMRI can help screen for early CSM and develop clinical surgical and rehabilitation treatment plans.

**Key points:**

• Diffusion MRI can differentiate between normal and early-stage cervical spondylotic myelopathy patients.

• Diffusion MRI has the ability to identify the extent of spinal cord involvement in cervical spondylotic myelopathy.

• Diffusion MRI enables the early screening of cervical spondylotic myelopathy and helps guide clinical treatment.

**Graphical Abstract:**

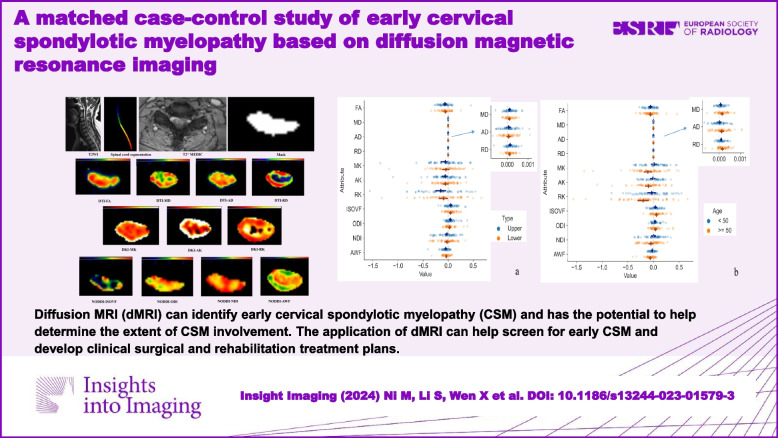

## Introduction

Cervical spondylotic myelopathy (CSM) is one of the most common forms of nontraumatic spinal cord injury in adults [1]. Early CSM usually has an insidious onset [1]; as the disease progresses, it may lead to irreversible damage to the spinal cord and symptoms such as sensory loss, spastic gait, delicate motor impairment, and urinary and faecal incontinence, eventually leading to disability [2]. Therefore, accurate identification and intervention for CSM patients as early as possible can reduce the adverse consequences caused by continuous progression of the disease.

Due to the mild clinical symptoms and diverse manifestations of early CSM, its diagnosis is difficult [3] and dependent upon the experience level of the clinician. MRI is a common examination method for the noninvasive preoperative, evaluation of CSM [4] and an important reference for formulating individualised treatment plans. However, early CSM does not have abnormal signals in the spinal cord or obvious spinal stenosis [5, 6], resulting in missed diagnoses in some early CSM patients [3]. Diffusion MRI (dMRI) leverages the diffusion behaviour of water molecules to characterise local microscopic tissue structural changes and has been used to evaluate CSM [7–9]. Current studies have proven that dMRI can noninvasively assess the severity of CSM and predict its prognosis before surgery [7, 10].

This 1:1 matched case–control study aims to explore the value of dMRI in identifying early CSM diagnosis through three dMRI models, diffusion tensor imaging (DTI), diffusion kurtosis imaging (DKI), and neurite orientation dispersion and density imaging (NODDI), and to explore the changes in the cervical spinal cord adjacent to uncompressed segments in patients with CSM.

## Materials and methods

This study was approved by the ethics committee of Peking University Third Hospital. Written informed consent was obtained from all the participants prior to the enrollment of this study.

### Patients

Between January 2021 and April 2023, patients with a clinical diagnosis of CSM were prospectively enrolled, and paired volunteers were recruited to undergo MRI examination. Volunteer recruitment was performed after CSM patients that met the inclusion conditions were recruited. The matching criteria for volunteers and CSM patients were that the age difference between the two was no more than five years, and the volunteers’ dMRI scan segments were consistent with those of the matched CSM patients. Volunteers and CSM patients were divided into a < 50-year-old group and a ≥ 50-year-old group. Due to scanning time limitations, the segment with the most severe compression was scanned if multiple segments were simultaneously involved (a total of 18 CSM patients).

**Fig. 1:**
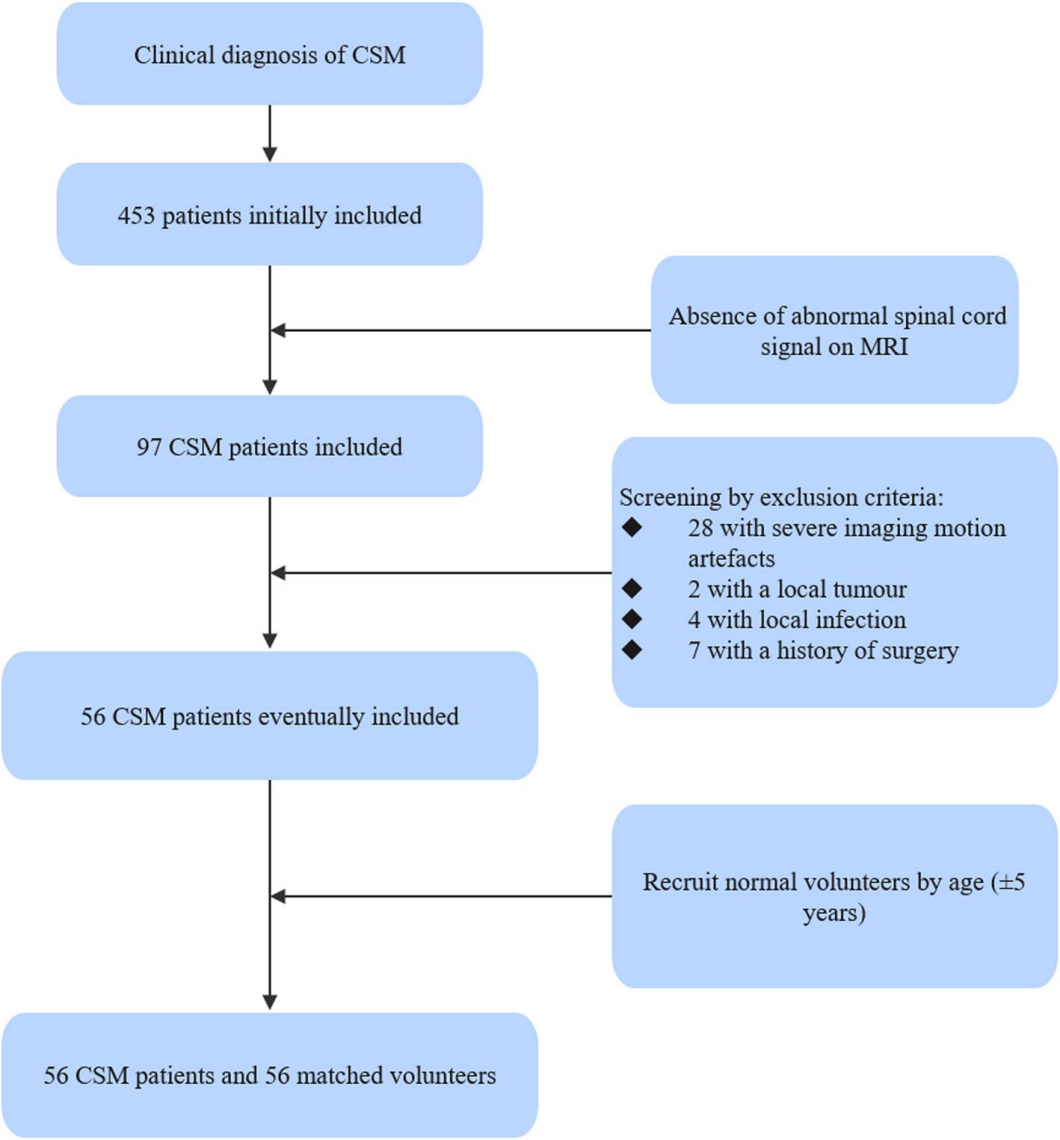
Schematic illustration of the flowchart indicating the baseline patient characteristics

Inclusion criteria for patients with CSM are as follows: (1) diagnosed with CSM by an orthopaedist (L.J., with 30 years of experience); (2) no abnormal signal within the spinal cord found in the MRI examination (evaluated by H.Y., with 35 years of experience). Inclusion criteria for volunteers are as follows: (1) clinical examination results not meeting a diagnosis of CSM; (2) no abnormal MRI signals observed in the spinal cord. Exclusion criteria are as follows: (1) severe image motion artefacts (assessed by M.N., with 8 years of experience); (2) CSM due to ossification of the posterior longitudinal ligament (OPLL) of the cervical spine; (3) previous spinal cord surgery, spinal cord trauma, spinal cord tumours, tuberculosis, and autologous history of immune diseases. The detailed inclusion and exclusion process is shown in Fig. 1.

### MRI scanning

All patients were scanned with a MAGNETOM Prisma 3.0T MRI (Siemens Healthcare). The imaging protocol comprised a sagittal turbo spin-echo (TSE) T1-weighted (T1WI) sequence, a T2-weighted imaging (T2WI) sequence, a transverse T2*-weighted multi-echo gradient echo sequence (multi-echo data image combination, MEDIC), and dMRI sequence (including DTI, DKI and NODDI) obtained by the zoomed imaging technique with parallel transmission (ZOOMit) technology (small field of view acquisition technology). The scanning parameters are shown in Table 1.

**Table 1:** Detailed parameters of MRI scans in this study

Sequence	Orientation	Repetition time (TR)	Echo time (TE)	Flip angle	Field of view (FOV)	Slice thickness	Spacing between slices	Diffusion-encoded directions
T1WI	Sagittal	4000 msec	8.5 msec	160°	280 × 280	3 mm	3.5 mm	/
T2WI	Sagittal	4350 msec	120 msec	150°	280 × 280	3 mm	3.5 mm	/
T2^a^ MEDIC	Transverse axial	400 msec	18 msec	30°	140 × 190	4 mm	4.4 mm	/
dMRI	Transverse axial	2000 msec	85 msec	90°	145 × 49	3 mm	3.3 mm	64 diffusion-encoded directions^a^

^a^ five acquisitions at b = 0 s/mm^2^ and two acquisitions at b = 800, 1600, and 2400 s/mm^2^

### dMRI data processing

**Fig. 2:**
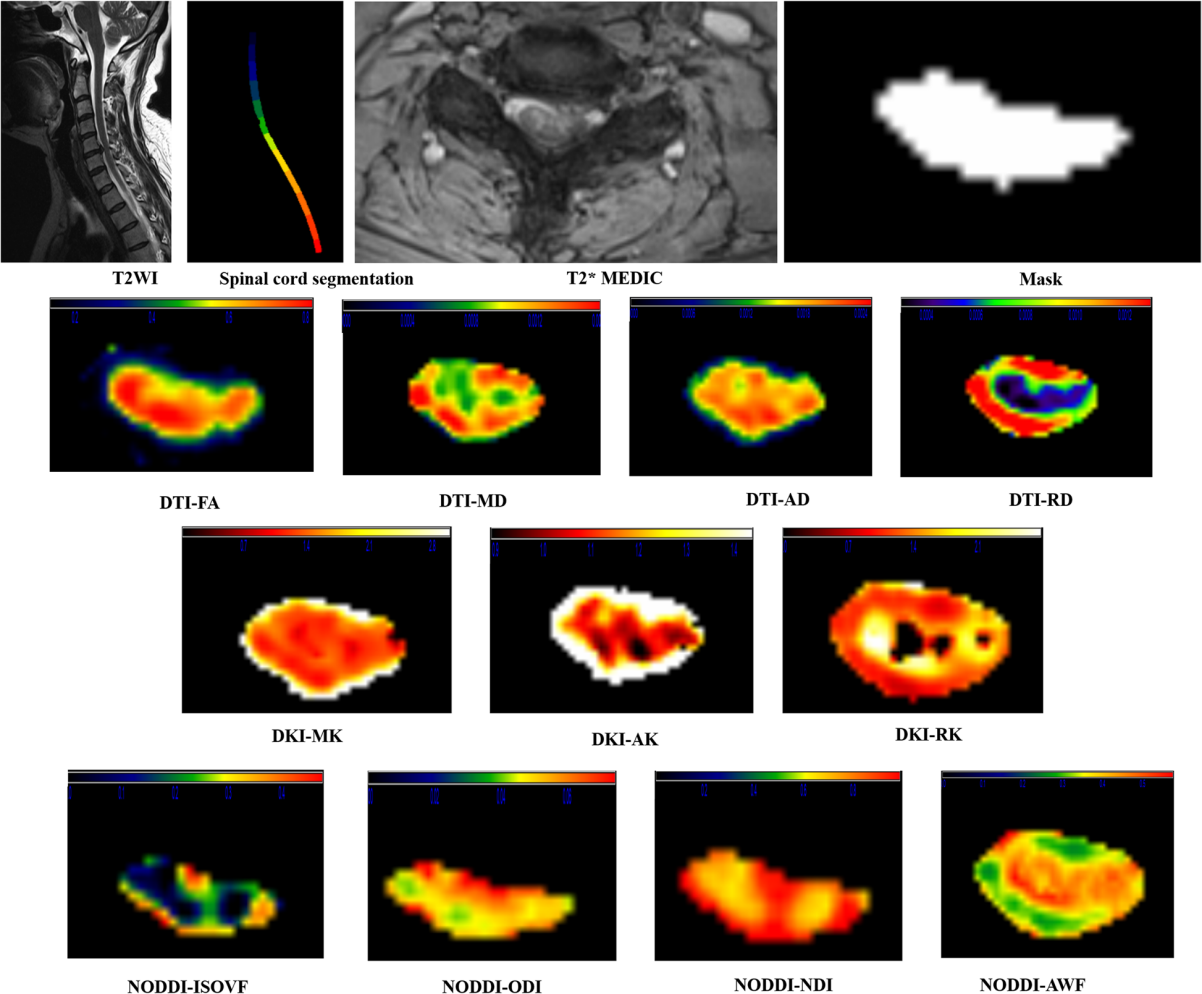
Automatic segmentation results and different dMRI variable colour maps based on sagittal T2WI and transverse T2*MEDIC. This patient was diagnosed with CSM due to C5–C6 cervical disc herniation compressing the spinal cord. The first row is the result of automatic spinal cord segmentation on T2WI images through SCT software and the ROI obtained based on automatic segmentation of transverse T2*MEDIC images. Rows 2-4 represent the colour maps of different DTI, DKI, and NODDI variables, respectively

The bias, motion, inhomogeneity and, eddy-current corrections were performed with Spinal Cord ToolBox (SCT, https://github.com/neuropoly/spinalcordtoolbox) and Fsleyes (https://pypi.org/project/fsleyes/) software packages. Regions of interest (ROIs) were obtained after automatically identifying spinal cord segments and segmenting the spinal cord through SCT software packages. The dMRI parameters were included the DTI-based fractional anisotropy (FA), mean diffusivity (MD), axial diffusivity (AD), radial diffusivity (RD), DKI-based mean kurtosis (MK), axial kurtosis (AK), radial kurtosis (RK), and NODDI-based isotropic volume fraction (ISOVF), orientation division index (ODI), neural density index (NDI), and anisotropic water fraction (AWF). The automatic segmentation results and the colour maps of different dMRI variables are illustrated in Fig. 2.

### dMRI and early CSM

The lesion area and adjacent uncompressed area on all dMRI scans were determined by two radiologists (radiologist 1, M.N.; radiologist 2, S.L., with 6 years of experience each), and when discrepancies occurred, a third, more senior radiologist (radiologist 3, H.Y.) determined the final result. If the lesion area and/or the adjacent uncompressed area spanned multiple layers on the scanned images, the average value of all included images was taken as the final parameter value.

The paired *t*-test was used to compare different dMRI parameters between groups, and dMRI parameters with significant differences were obtained. The variance inflation factor (VIF) was used to evaluate any multicollinearity that might be present among the dMRI parameters. Starting from all parameters through the backwards stepwise selection method, the parameters with the highest VIF values were gradually removed until the VIF values of the remaining parameters do not exceed 10. The filtered dMRI parameters were analysed using logistic regression to obtain significant parameters distinguishing volunteers from early CSM patients.

When researching the adjacent upper and lower uncompressed areas of CSM patients, the difference method was used to eliminate the inherent differences in dMRI parameters of different spinal cord segments [11]. The corresponding values of the matched volunteers were subtracted from the dMRI values of the CSM patients in the paired data to obtain the difference value (for example, FA_CSM_-FA_Volunteer_=FA_D-value_). Finally, the univariate t-test was used to verify whether the mean of the difference was equal to zero when analysing CSM to evaluate the differences between patients and volunteers in adjacent uncompressed areas.

## Statistical analysis

All statistical analyses were performed using Python software (version 3.6.0; Python Software Foundation). The paired t-test was used to analyse differences in dMRI parameters between groups. The VIF is used to evaluate multicollinearity between dMRI parameters. When the VIF value between parameters was < 10, they were considered to demonstrate no significant multicollinearity, whereas larger VIF values indicated stronger multicollinearity. Logistic regression analysis was used to determine the dMRI parameters that could significantly differentiate between normal subjects and CSM patients, and correlation analysis was performed. The univariate t-test was used to calculate the difference in dMRI parameters between CSM patients and volunteers in the adjacent upper and lower uncompressed spinal cord. A *p*-value smaller than 0.05 was considered to indicate statistical significance.

## Results

**Fig. 3:**
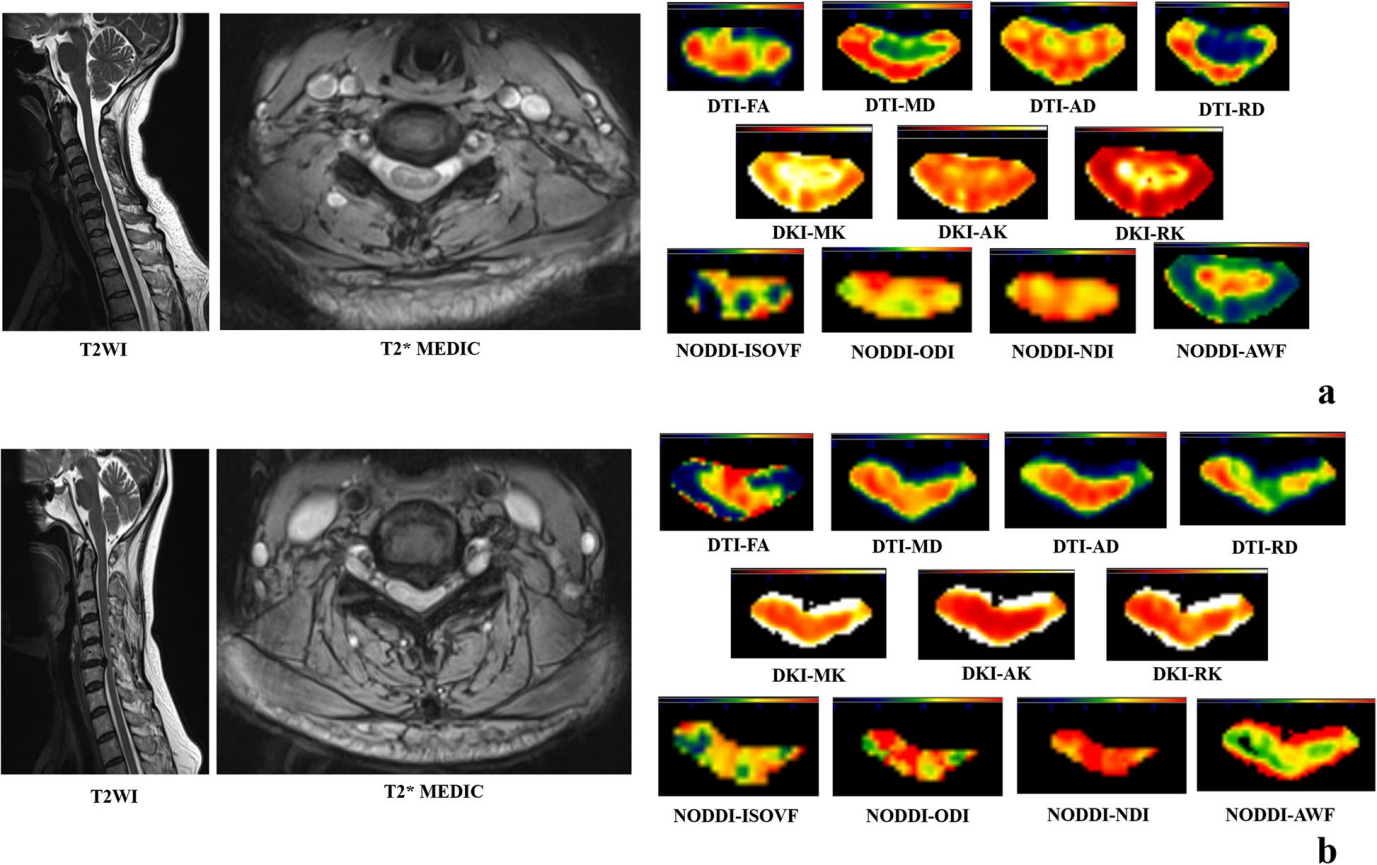
Conventional imaging and dMRI variable colour maps of patients with CSM occurring at C5–C6 and matched volunteers. **a** The volunteers and **b** the matched CSM patients. Rows 1–3 of the right picture represent the colour maps of different DTI, DKI, and NODDI variables, respectively. The colour maps of the variable are manually adjusted to show the clearest image contrast. In **a**, the FA value is 0.0955, the MD value is 0.0001, the AD value is 0.0003, the RD value is 0.0002, the MK value is 0.2163, the AK value is 0.0753, the RK value is 0.4833, the ISOVF value is 0.1388, and the ODI value is 0.0129. The NDI value is 0.1306 and the AWF value is 0.0525; in **b**, the FA value is 0.0892, the MD value is 0.0001, the AD value is 0.0002, the RD value is 0.0002, the MK value is 0.2155, the AK value is 0.0701, and the RK value is 0.0721. The ISOVF value is 0.1616, the ODI value is 0.0722, the NDI value is 0.1211, and the AWF value is 0.01012

A total of 56 early CSM patients and 56 matched volunteers were included in the study, forming a total of 56 case-matched groups (1:1 matching). The basic clinical information and spinal location distribution are detailed in Table 2. Conventional imaging and dMRI variable colour maps of paired volunteers and CSM patients are shown in Fig. 3.

**Table 2:** Basic clinical information and spinal location distribution of CSM patients and volunteers included in the matched case-control study

Group	Location	Subject (*n*)	Age (years, mean±SD)	Sex (*n*)^a^
CSM	C2–C3	0	/	/
	C3–C4	6	56.67 ± 11.02	4 M and 2 F
	C4–C5	16	55.00 ± 15.60	10 M and 6 F
	C5–C6	26	49.08 ± 11.31	16 M and 10 F
	C6–C7	8	54.63 ± 12.60	5 M and 3 F
Volunteers	C2–C3	0	/	/
	C3–C4	6	54.17 ± 11.92	4 M and 2 F
	C4–C5	16	51.63 ± 9.37	9 M and 7F
	C5–C6	26	49.03 ± 10.91	12 M and 14 F
	C6–C7	8	52.88 ± 5.82	5 M and 3 F

^a^: *M*, male; *F*, female

**Fig. 4:**
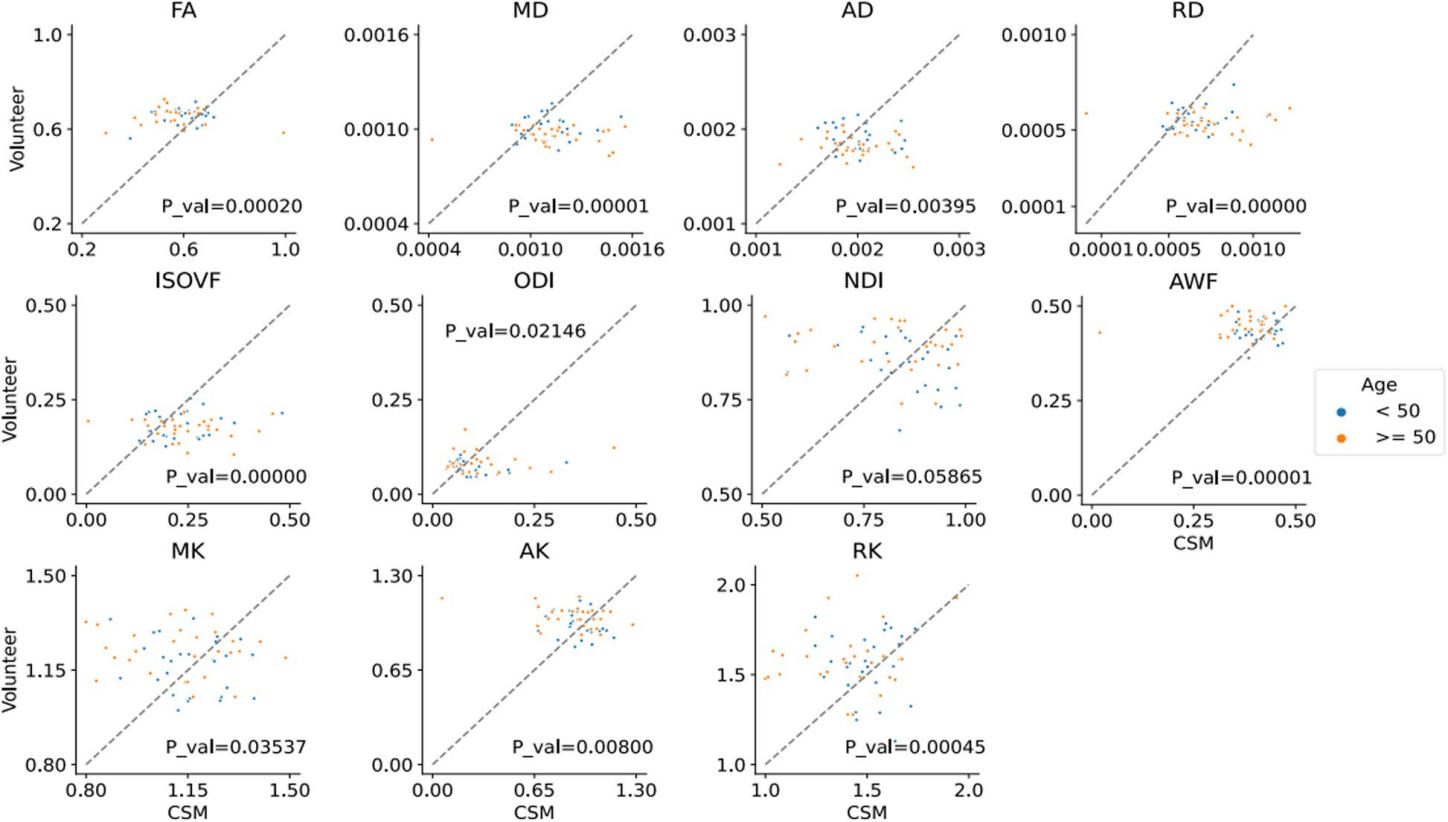
Distribution of 11 parameters obtained by dMRI. The participants are grouped according to age (< 50 years and ≥ 50 years), where the first row represents DTI parameters, the second row represents NODDI parameters, and the third row represents DKI parameters. The *p*-value in the lower left corner represents the result of the paired *t*-test; a *p*-value less than 0.05 represents statistical significance. All dMRI parameters except NDI and MK showed significant differences between CSM patients and normal volunteers

A total of 11 parameters were obtained from dMRI scans in this study. The distribution of the parameters and paired *t*-test results are shown in Fig. 4. The paired *t*-test results showed that except for MK and NDI, the remaining nine parameters had significant differences between early CSM patients and volunteers. Therefore, further correlation analysis was needed to identify the parameters with the strongest correlation. The multicollinearity of all dMRI parameters was calculated through the VIF, and the backwards stepwise selection method was used for screening. Finally, AD (VIF = 5.98), RD (VIF = 6.32), RK (VIF = 2.07), ODI (VIF = 4.93), NDI (VIF = 2.93) and AWF (VIF = 1.76) were identified as having no significant multicollinearity. By performing logistic regression on the above-screened dMRI parameters, the results showed that the ODI and AWF parameters were significantly correlated, with ODI showing a significant positive correlation (*r* = 2.12, *p* = 0.035) and AWF showing a significant negative correlation (*r* = −0.98, *p* = 0.015). The results of the logistic regression analysis are detailed in Table 3.

**Table 3:** Logistic regression results after VIF filtering for parameter multicollinearity

Parameters	Coefficient^b^	Standard error	*Z* value	*p*-value	95% CI^c^
AD	1.39	0.77	1.80	0.07	[−0.122, 2.907]
RD	0.10	0.79	0.12	0.9	[−1.449, 1.643]
RK	0.17	0.35	0.48	0.63	[−0.512, 0.846]
ODI	2.12	1.01	2.10	0.04	[0.145, 4.098]
NDI	−0.12	0.51	−0.24	0.81	[−1.117, 0.871]
AWF	−0.98	0.41	−2.42	0.02	[−1.775, −0.187]
Age^a^	−0.72	0.51	−1.41	0.16	[−1.713, 0.279]

^a^: represents no significant difference between the < 50 years old and ≥ 50 years old groups; ^b^: positive numbers represent a positive correlation, negative numbers represent a negative correlation; ^c^: represents the 95% confidence interval of the regression coefficient

**Fig. 5:**
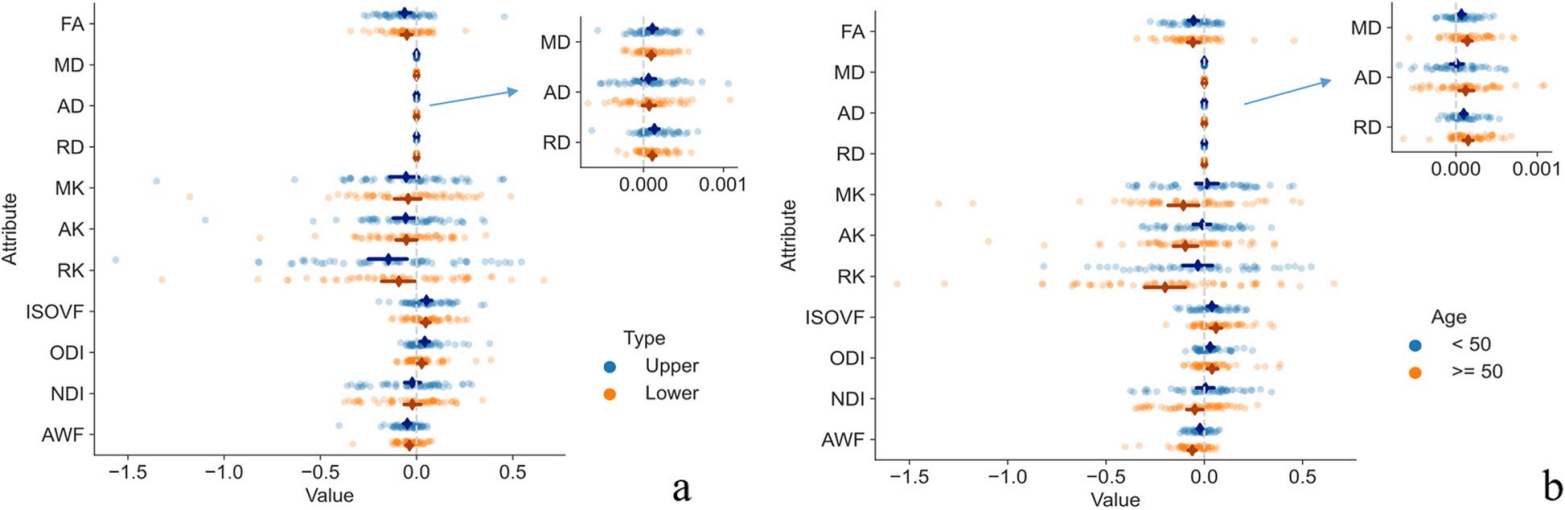
Difference map of the parameters in adjacent uncompressed cervical spinal cord segments in patients with early CSM and volunteers. All differences were obtained by subtracting the values of the corresponding volunteers from those of the CSM patients. Panel **a** shows the dMRI parameter distribution of the uncompressed areas above and below the adjacent segments of CSM lesions; **b** shows the dMRI parameter distribution of the uncompressed areas above and below the adjacent segments of CSM lesions grouped according to age

In the analysis of the adjacent uncompressed spinal cord of early CSM patients, the difference results obtained after subtraction between CSM patients and volunteers are shown in Fig. 5. The univariate *t*-test results showed that in patients with CSM, FA (*p* < 0.001), MD (*p* < 0.001), RD (*p* < 0.001), RK (*p* = 0.007), ISOVF (*p* = 0.001), ODI (*p* < 0.001) and AWF (*p* < 0.001) were significantly different between volunteers in the adjacent upper uncompressed areas. In the adjacent lower uncompressed areas, FA (*p* < 0.001), MD (*p* < 0.001), RD (*p* < 0.001), ISOVF (*p* < 0.001), ODI (*p* = 0.023), and AWF (*p* < 0.001) were significantly different. In the age-grouped subanalyses, for those aged < 50 years, FA (*p* < 0.001), MD (*p* = 0.003), RD (*p* < 0.001), ISOVF (*p* = 0.002), ODI (*p* = 0.002), and AWF (*p* = 0.001) were significantly different between CSM patients and volunteers, while in those aged ≥ 50 years, all parameters were significantly different (all *p* < 0.05). All age groups had significant differences in the upper and lower regions in FA, MD, RD, ISOVF, ODI, and AWF. The detailed results of the univariate *t*-test are shown in Table 4.

**Table 4:** Univariate *t*-test results of the adjacent cervical spinal cord in patients with early CSM based on location and age grouping (< 50 and ≥ 50 years)

Parameters	Upper		Lower		Age < 50 years		Age ≥ 50 years	
	*t* value	*p-*value	*t* value	*p*-value	*t* value	*p*-value	*t* value	*p*-value
FA	−4.05	**< 0.001**	−3.77	**< 0.001**	−4.42	**< 0.001**	−3.62	**0.001**
MD	3.76	**< 0.001**	4.09	**< 0.001**	3.12	**0.003**	4.63	**< 0.001**
AD	1.50	0.141	1.71	0.094	0.31	0.762	2.68	**0.010**
RD	4.67	**< 0.001**	4.72	**< 0.001**	4.52	**< 0.001**	4.99	**< 0.001**
MK	−1.41	0.166	−1.24	0.219	0.44	0.659	−2.58	**0.013**
AK	−1.84	0.071	−1.97	0.054	−0.46	0.647	−2.93	**0.005**
RK	−2.82	**0.007**	−1.99	0.051	−0.82	0.416	−3.70	**< 0.001**
ISOVF	3.67	**0.001**	4.07	**< 0.001**	3.25	**0.002**	4.39	**< 0.001**
ODI	3.76	**< 0.001**	2.33	**0.023**	3.19	**0.002**	3.02	**0.004**
NDI	−1.05	0.297	−1.01	0.318	0.25	0.805	−2.33	**0.023**
AWF	−4.90	**< 0.001**	-4.16	**< 0.001**	−3.43	**0.001**	−5.74	**< 0.001**

## Discussion

In this work, CSM patients and volunteers were matched 1:1 according to spinal location and age through a matched case-control study, aiming to explore the value of dMRI in identifying early CSM patients and analysing spinal cord changes in adjacent uncompressed segments in CSM patients. As ODI increases, the probability of patients being diagnosed with CSM also increases; as AWF decreases, the probability of patients being diagnosed with CSM increases. Combining ODI and AWF can help identify early CSM. In the cervical cord of the adjacent upper and lower uncompressed segments of CSM patients, FA, MD, RD, ISOVF, ODI, and AWF significantly differed from those of volunteers among all ages.

Current studies have revealed significant differences in dMRI parameters between different ages and spinal cord segments, but no significant differences have been identified between sexes [12, 13]. Therefore, when performing early CSM analysis by dMRI, it is crucial to consider the differences in age and cervical spinal cord segments so that the evaluation results obtained after eliminating interfering factors are more accurate. Existing studies used DTI and DKI to diagnose early CSM [5, 14–16]; however, most studies did not include normal people as control groups and only used dMRI parameters of other cervical spinal cord segments for comparison. Only one study included normal volunteers as the control group [16], but it did not conduct a matched case-control study by segment and age, ignoring inherent differences within different segments and not accounting for age. Additionally, no studies have used the NODDI model. Compared with DTI and DKI, the NODDI model can more accurately characterise the direction and density of nerve fibre bundles and detect abnormalities therein more sensitively [17].

Early CSM is prone to be ignored, but as the disease develops, it will result in an extensive disease and economic burden. Therefore, establishing reasonable, noninvasive screening and diagnostic methods will help increase the detection rate of CSM. Existing research suggests that surgical treatment is better in individuals with mild CSM than in those with severe CSM [18–22]. Early diagnosis of CSM and adequate intervention will help prevent the continued progression of the disease and improve patient prognosis. The results of this study show that ODI is significantly elevated in the cervical spinal cord segments of CSM patients. The ODI is used to characterise the directional dispersion of nerve fibre bundles. This parameter takes values from 0 to 1; as the value increases, the direction of the nerve fibre bundles changes from completely consistent to completely dispersed [23], and so the ODI can show whether the nerve fibre bundles within the spinal cord have already undergone significant direction dispersion changes prior to the manifestation of MRI signals visible to the naked eye. We also found that the AWF is significantly reduced in CSM patients. The AWF is used to estimate the water content of axons and can be used to evaluate the integrity of white matter tracts. A smaller AWF indicates thinning of the axon wall and a decrease in the water content, suggesting that in the early stage of CSM, the axons are stretched and squeezed, resulting in damage to the axonal myelin sheath, thinning of the axonal wall, and even axonal rupture. Therefore, the ODI and AWF can identify early CSM and reflect its microscopic pathological, disease and developmental changes, helping clinicians identify CSM patients earlier.

According to the AOSpine North America and the Cervical Spine Research Society (CSRS) guidelines, for mild CSM, surgical intervention or supervised structured rehabilitation should be provided [24]. Currently, the main surgical treatment method for CSM is spinal decompression (including anterior, posterior, or combined approaches), and preoperative MRI examination is a critical reference for determining the scope of surgery and formulating rehabilitation treatment plans [25, 26]. This study compared the uncompressed cervical spinal cord lesions of CSM patients and volunteers and found that the dMRI parameters of CSM patients were significantly different from those of volunteers, which means that even if there is no compression, the spinal cord of adjacent segments is also affected by CSM, a change that cannot be detected with conventional imaging examinations. Although this study did not explore the specific extent of spinal cord involvement in patients with CSM, the results demonstrate that dMRI can assist in accurately identifying the extent of CSM involvement before surgery. Therefore, when performing surgical treatment, dMRI can help minimise damage to normal tissues and fully reveal the range of decompression to ensure that the affected spinal cord can be effectively covered to obtain better surgical treatment effects. We also identified multiple significantly different dMRI parameters in the upper and lower segments adjacent to the lesion between the groups, including ODI and AWF. Therefore, AWF and ODI can be used to screen for early CSM and help determine the scope of disease involvement to a certain extent.

## Limitations

(1) Only the most severe segment was scanned for CSM patients with multisegment involvement in this study. Since the dMRI scan uses a small field of view, multisegment scanning took a relatively long time. Therefore, in patients with multisegment CSM, there may be additive effects on dMRI parameters adjacent to uncompressed areas. (2) The changes in the cervical spinal cord adjacent to the intervertebral segments of CSM lesions were not studied, and the extent of CSM involvement was not explored, which may also be affected by factors such as the duration of CSM. (3) This study excluded patients with CSM caused by OPLL compression. Since compression of the OPLL can be very extensive (potentially involving the cervical and thoracic spine simultaneously) and the surgical methods are different, no relevant studies were conducted.

## Conclusion

dMRI can noninvasively identify patients with early CSM and has the potential to identify the extent of CSM lesions involving the cervical spinal cord. The NODDI-based ODI and AWF are of great value in distinguishing normal people from early CSM patients and in determining the extent of disease involvement. The results of this study can help identify early CSM patients, allow more early CSM patients to benefit from treatment, and help clinicians better understand the disease mechanism and formulate individualised treatment plans.

## Data Availability

All imaging and clinical data are stored in the database of the Radiology Department of Peking University Third Hospital. Please get in touch with Peking University Third Hospital for data acquisition.

## Abbreviations

AD: Axial diffusivity
AK: Axial kurtosis
AWF: Anisotropic water fraction
CSM: Cervical spondylotic myelopathy
DKI: Diffusion kurtosis imaging
dMRI: Diffusion MRI
DTI: Diffusion tensor imaging
FA: Fractional anisotropy
ISOVF: Isotropic volume fraction
MD: Mean diffusivity
MK: Mean kurtosis
NDI: Neural density index
NODDI: Neurite orientation dispersion and density imaging
ODI: Orientation division index
OPLL: Ossification of the posterior longitudinal ligament
RD: Radial diffusivity
RK: Radial kurtosis
VIF: Variance inflation factor
ZOOMit: Zoomed imaging technique with parallel transmission
